# Supplementary material for: High Frequency Hysteresis Losses on γ-Fe2O3 and Fe3O4: Susceptibility as a Magnetic Stamp for Chain Formation
Source: Nanomaterials (Basel). 2018 Nov 24;8(12):970. doi: 10.3390/nano8120970 (PMC6315427; doi:10.3390/nano8120970)
Supplement: Supplementary file 1 [file nanomaterials-08-00970-s001.pdf]

Article

High Frequency Hysteresis Losses on γ-Fe_2O_3 and Fe_3O_4: Susceptibility as a Magnetic Stamp for Chain Formation

Irene Morales [1], Rocio Costo [2], Nicolas Mille [3], Gustavo B. da Silva [2], Julian Carrey [3], Antonio Hernando [1,4], and Patricia de la Presa [1,4,*]

[1] Instituto de Magnetismo Aplicado (UCM-ADIF-CSIC), P.O. Box 155, Las Rozas, 28230 Madrid, Spain; irenemorales@ucm.es (I.M.); antonio.hernando@externos.adif.es (A.H.)
[2] Instituto de Ciencia de Materiales de Madrid/CSIC, Sor Juana Inés de la Cruz 3, 28049 Madrid, Spain; rocio.costo@gmail.com (R.C.); gusbezerra@ufrrj.br (G.B.d.S.)
[3] Université de Toulouse, INSA, UPS, Laboratoire de Physique et Chimie des Nano-Objets (LPCNO), 135 Avenue de Rangueil, F-31077 Toulouse, France and CNRS, UMR 5215, LPCNO, F-31077 Toulouse, France; mille@insa-toulouse.fr (N.M.); julian.carrey@insa-toulouse.fr (J.C.)
[4] Departamento de Física de Materiales, Universidad Complutense de Madrid, 28048 Madrid, Spain
* Correspondence: pmpresa@ucm.es; Tel.: +34-91-300-71-73

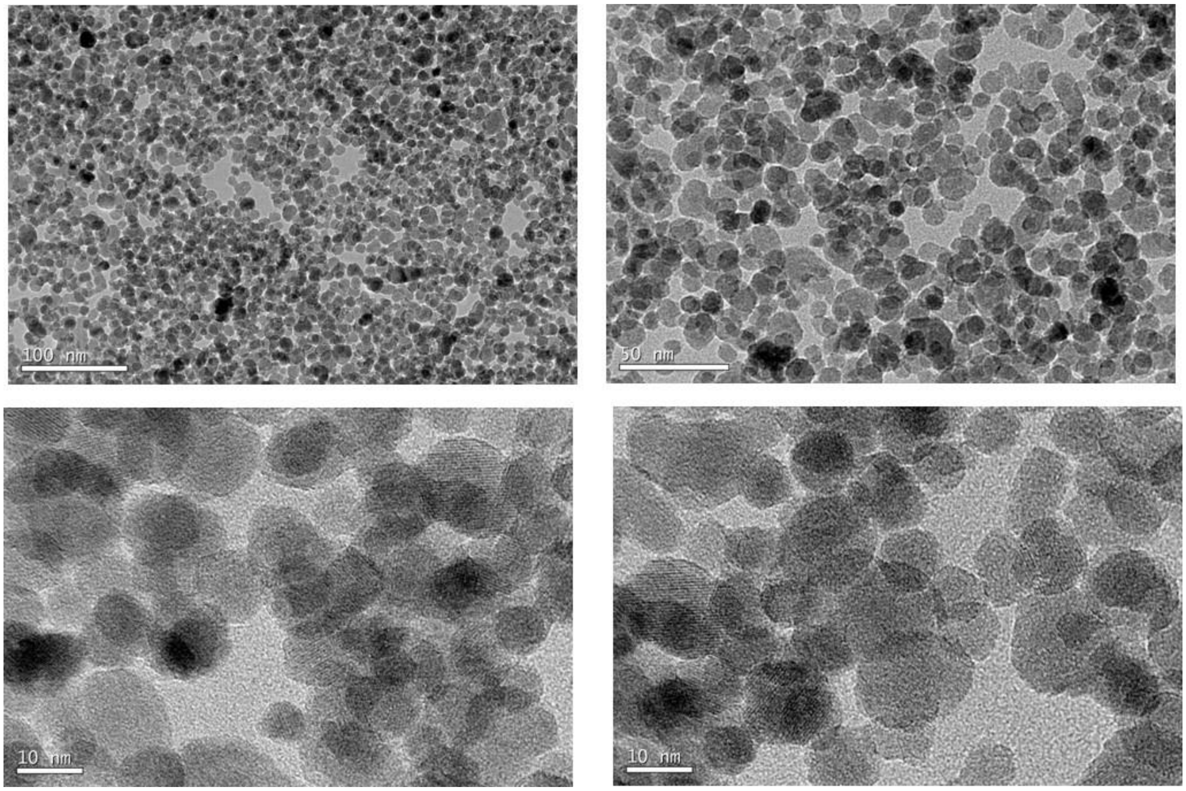

Figure S1. (**a**) TEM images γ-Fe2O3-12nm at different augmentations.

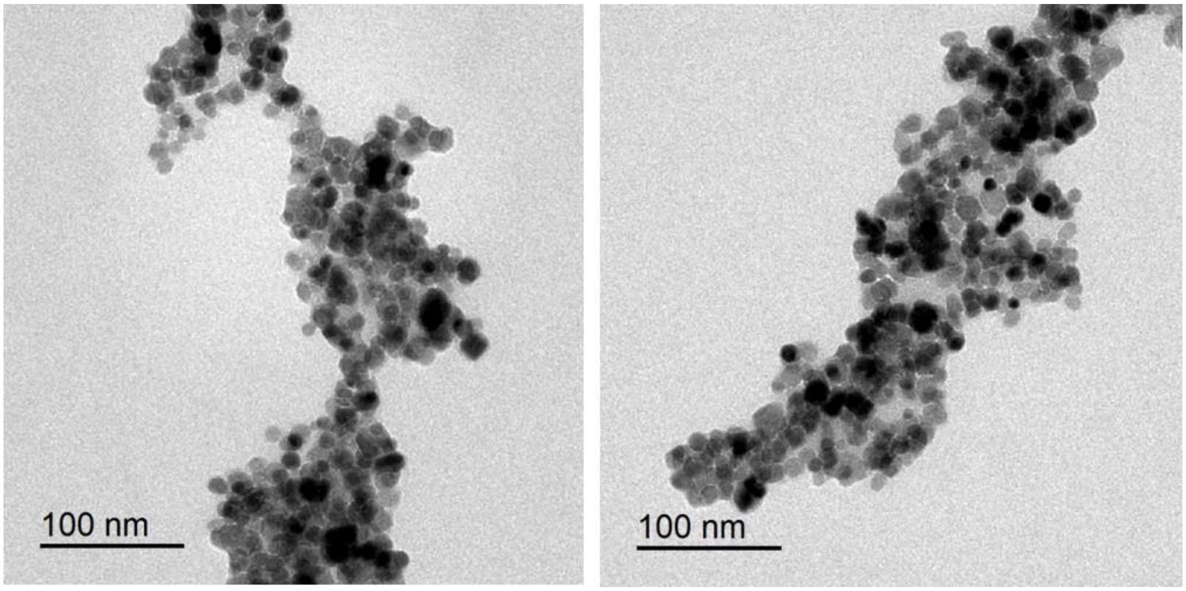

Figure S1. (**b**) TEM images γ-Fe2O3-14nm.

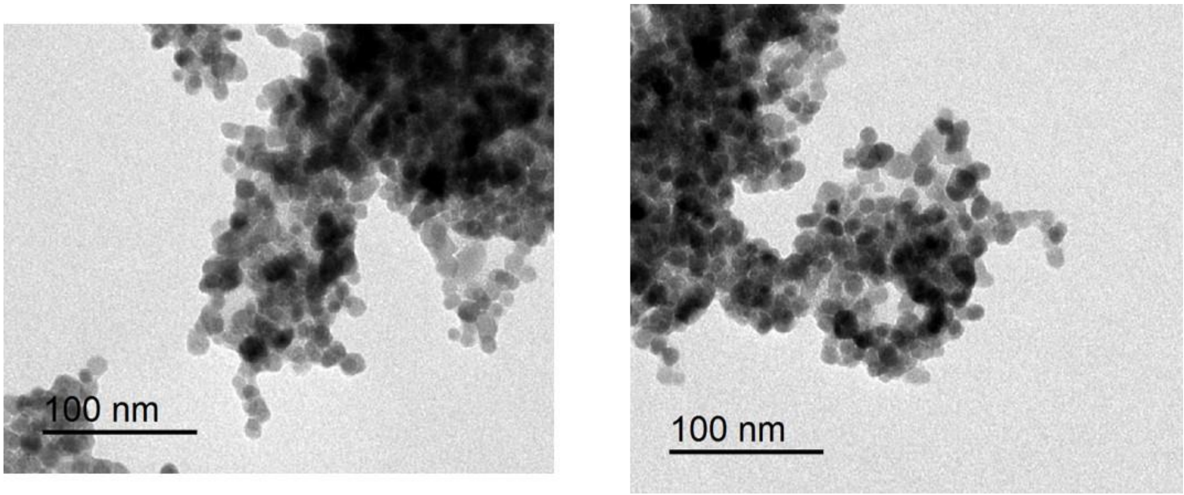

Figure S1. (**c**) TEM images Fe3O4-14nm.

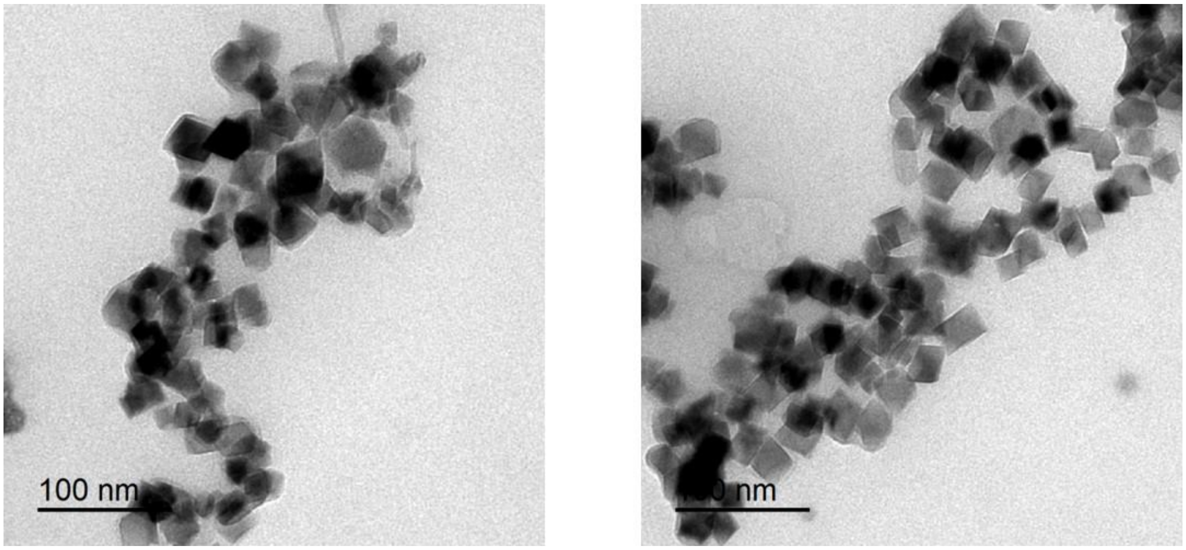

Figure S1. (**d**) TEM images Fe3O4-35nm.

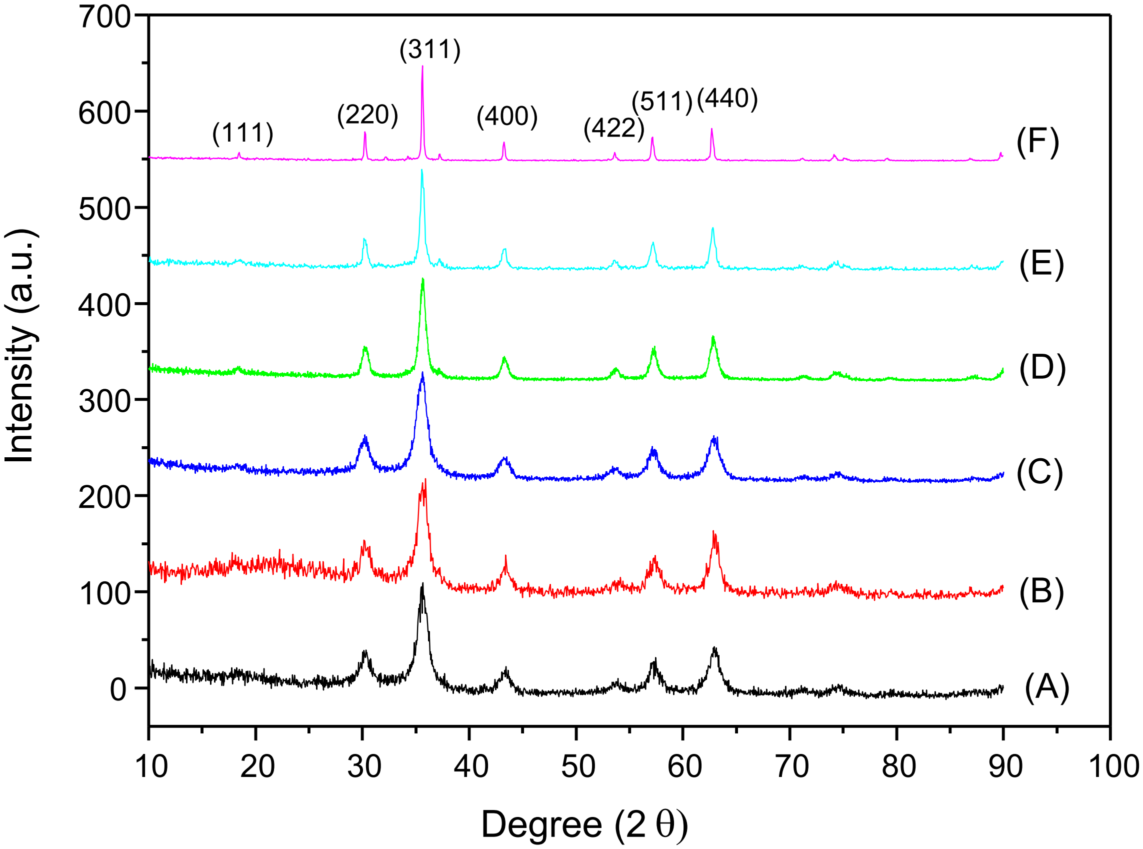

Figure S2. X-ray diffraction patterns for the maghemite and magnetite nanoparticles: (**A**) γFe_2O_3-6nm, (**B**) γFe_2O_3-8nm, (**C**) γFe_2O_3-12nm, (**D**) γFe_2O_3-14nm, (**E**) Fe_3O_4-35nm, and (**F**) Fe_3O_4-350nm.

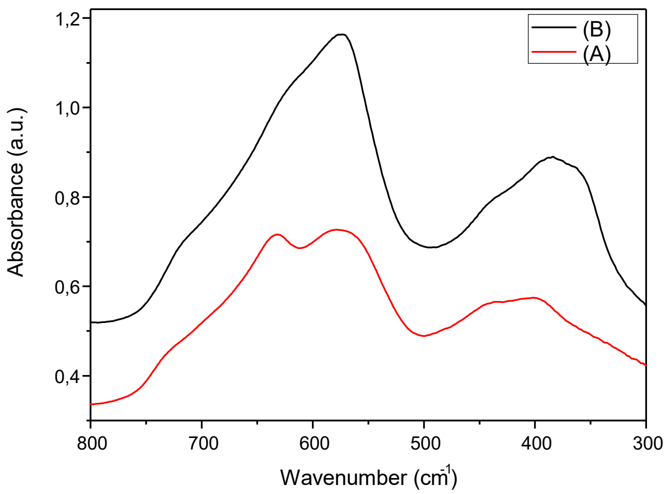

Figure S3. FTIR spectra of samples (**A**) γFe_2O_3-8nm, and (**B**) Fe_3O_4-14nm between 800 and 300 cm^{-1} which correspond to Fe-O vibrations.

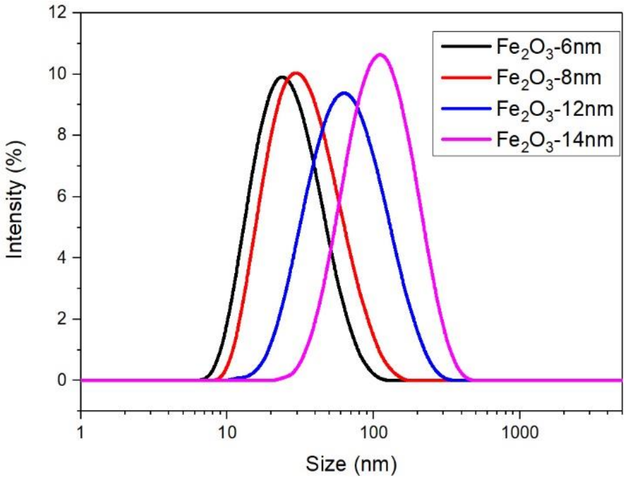

Figure S4. Hydrodynamic sizes D_h at pH7 for γ-Fe_2O_3 nanoparticles.

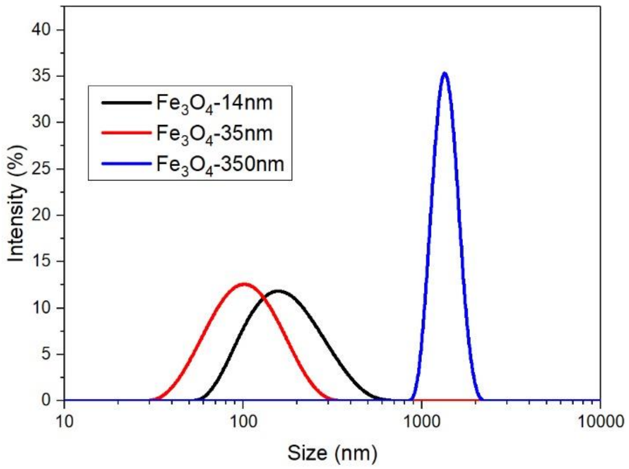

Figure S5. Hydrodynamic sizes D_h at pH7 for Fe_3O_4 nanoparticles.

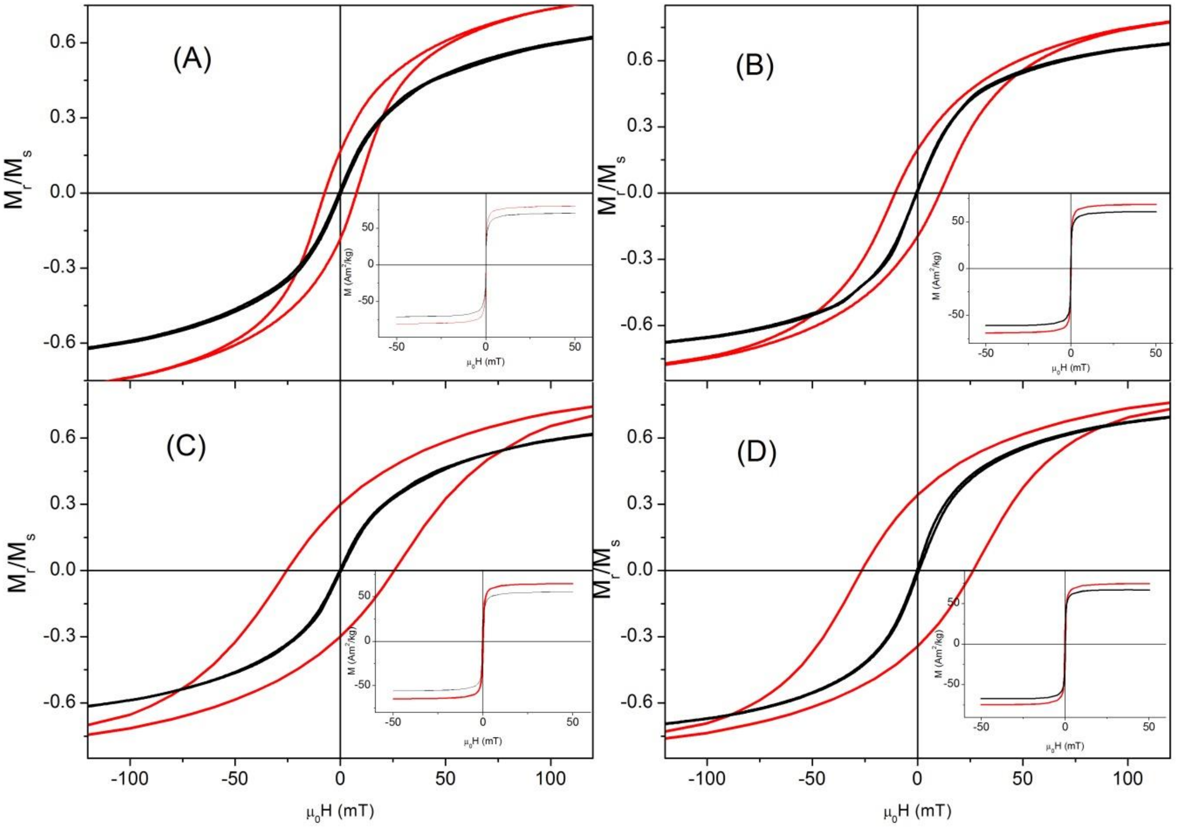

Figure S6. Hysteresis loops at 10 K (red) and 300 K (black) for samples (**A**) γFe_2O_3-6nm, (**B**) γFe_2O_3-8nm, (**C**) γFe_2O_3-12nm, and (**D**) γFe_2O_3-12nm.

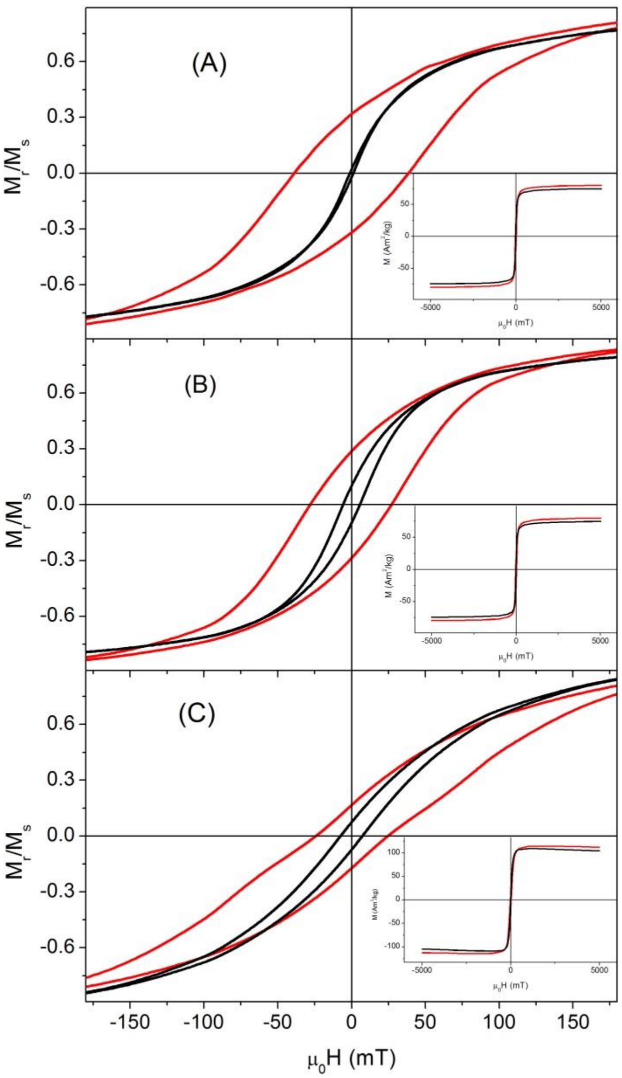

Figure S7. Hysteresis loops at 10 K (red) and 300 K (black) for samples (**A**) Fe_3O_4-14nm, (**B**) Fe_3O_4-35nm, and (**C**) Fe_3O_4-350nm.
